# Are Physician Associates Less-defined Force Multipliers? Comparative Role Definition of Physician Associates within the Hierarchy of Medical Professionals

**DOI:** 10.7759/cureus.6469

**Published:** 2019-12-26

**Authors:** Bilal Haider Malik, Ratna Krishnaswamy, Safeera Khan, Deepti Gupta, Ian Rutkofsky

**Affiliations:** 1 Internal Medicine, California Institute of Behavioral Neurosciences and Psychology, Fairfield, USA; 2 Research, California Instititute of Behavioral Neurosciences and Psychology, Fairfield, USA; 3 Family Medicine, California Institute of Behavioral Neurosciences and Psychology, Fairfield, USA; 4 Reproductive Medicine, Saint Mary's Hospital, Manchester, GBR; 5 Psychiatry, California Institute of Behavioral Neurosciences and Psychology, Fairfield, USA

**Keywords:** physician assistant, physician associate, assistant physician, mid-level practitioner, mid-level professional

## Abstract

Medical field has changed considerably with pressures added on mainly by soaring costs, a decline in the workforce strength and patient expectations. The solution that healthcare systems have come up with is the induction of physician associates (PAs) into the workforce. We aim to compare and contrast PAs with other members of the healthcare teams such as nurses, doctors and assistant physicians to demonstrate the vital role PAs play in the current healthcare environment. With the increased patient load and shrinking medical workforce, there are fears that chronic disease management in primary and secondary care will be threatened. Therefore, health policymakers thought of developing a new mid-level practitioner role (such as PAs, ANPs and APs) that will augment physicians to cater for ever-growing complex medical needs of the patients. The role of PAs is comparable to many healthcare professionals, and one can say that the success of PA programmes has paved the way for the development of different other mid-level practitioner development initiatives. All these roles are there to support primary and secondary care physicians in both inpatient and outpatient settings in helping the patients. PAs are a force multiplier within the healthcare sector and can be seen as a valid solution to staff shortages faced by the healthcare systems around the world. We recommend further studies looking into different aspects of the role of a PA that could further provide our readers with clarity with regard to PAs.

## Introduction and background

“Wherever the art of Medicine is loved, there is also a love of Humanity.”― Hippocrates

In the United States alone, there were 115,547 practicing physician assistants or physician associates (PAs) in 2018 [1]. If you add on the numbers globally, it is a major workforce to reckon with. They are working along the spectrum of medical and surgical specialties around the world and adding making invaluable contributions to the healthcare system.

Medical field has changed considerably over the past few years with pressures added to mainly by soaring costs, a decline in the workforce strength and patient expectations [2-3]. Even in the western healthcare systems which are considered to be a benchmark, like in the United States, there will be a shortage of surgeons and physicians in forthcoming couple of decades [4-10]. The solution that some of these healthcare systems have come up with is the induction of PAs into their workforce. For example, physician assistants have been a part of the American Healthcare system since 1967 and have been providing their services across the fields of surgery and medicine [4,11]. During the past decade, the demand and supply of PAs has grown considerably, and hence, we can deduce that PAs will be a major healthcare cohort providing care to our ever-increasing and aging population [4,6,12]. Therefore, in the minds and plans of medical workforce planners, PAs are a workforce categorized as a force multiplier, a cohort that augments the effectiveness of the care delivery considerably.

**Figure 1:**
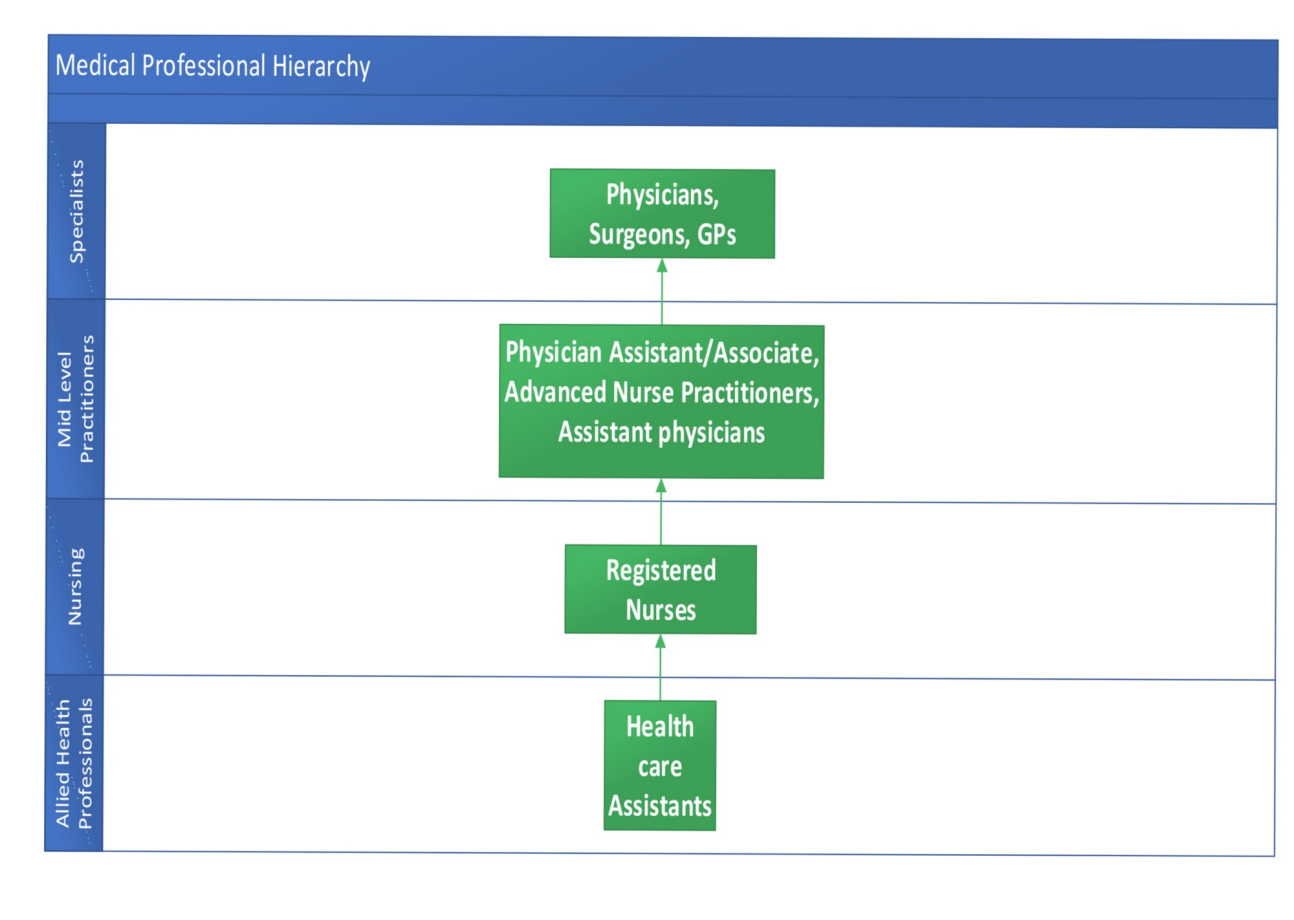
Medical professional hierarchy GP, general practitioner

There is a degree of consensus among the healthcare staff with regard to the role and responsibilities of the PAs in the western healthcare systems, especially the United States [13]. But it has also been observed that even in some of the western healthcare systems, there is a degree of lack of clarity with regard to the PA cohort’s roles and responsibilities as they are trying to constitute regulatory bodies to regulate this emerging profession [13]. There have also been queries surrounding the curriculum development and outcomes, and the work is underway to streamline this process [14]. The issue of curriculum delivery using the current medical educator faculty has also been raised, and this has been realized that present academic workforce must grow and needs to be sufficiently trained, to deal with task at hand which is going to add to indirect costs of running, current and future programs requiring further funding [15]. Work is also underway to best support all the stakeholders involved during the introduction and integration of this workforce in the healthcare systems around the world. Figure 1 explains the hierarchy of medical professionals within a team.

 

Hence, we aim to produce a piece of work that encompasses relevant issues with regard to the emerging profession of PAs and provides an insight into healthcare systems across the globe into the world of PAs. We have reviewed the relevant pieces of academic work using the PubMed search platform and produced a review paper to help and guide our readers regarding the PA’s role as a force multiplier.

## Review

Results

There is expected to be a shortage of primary care physicians in the United States (around 49,000) by 2030 because of the retiring workforce. With the increased patient load and shrinking medical workforce, there are fears that chronic disease management in primary and secondary care will be threatened. Therefore, health policymakers thought of developing a new mid-level practitioner role that will augment physicians to cater to the ever-growing complex medical needs of the patients. With the US being the leader in the development of the PA role, other healthcare systems are following the same principles to develop PA training programmes. The Health Education England (HEE) alone aims to induct around 1000 PAs in primary care by 2020. Many doctors and members of the public recognise PAs and NPs as a core element in improving the performance of primary care. Hence PA-primary physician partnership has been flourishing for over five decades now and continues to do so. The presence of PAs in secondary inpatient care also tends to increase doctor’s productivity as PAs are able to manage specific tasks in a more suitable way. The introduction of the PA workforce into outpatient settings has also allowed the doctors to focus more on patient care whilst increasing the number of patients seen in the outpatient settings with better outcomes, and this has translated into improved productivity and profits. In addition to the PAs, there has been a development of a new kind to practitioners in certain states of the US with Missouri being the pioneering state; these practitioners are called assistant physicians. Requirements for licensure for assistant physicians include graduation from a recognized medical school, citizen or a legal resident of the US, passed USMLE step 1 and step 2, not accomplished the completion of residency and proficiency in the English language. Requirements for practicing for assistant physicians include; Collaborative agreement with a sponsoring physician, 30-day internship with the sponsoring physician, work within 50-100 miles of the sponsoring physician and this physician must undertake a review of 10% of the Assistant physician’s charts. Therefore, the role of PAs is comparable to many healthcare professionals and one can say that the success of PA programmes has paved the way for the development of different other mid-level practitioner development initiatives. All these roles are there to support primary and secondary care physicians in both inpatient and outpatient settings to help the patients.

Discussion

Nurses and PAs

The Incorporation of Advanced practice providers (APPs) into healthcare systems around the world can increase access to healthcare for public in need and reduce care costs [16]. Advanced practitioners are PAs, clinical nurse specialists (CNSs), advanced practice registered nurses (APRNS), certified registered nurse anaesthetists (CRNAs) and certified nurse midwives (CNMs) [16]. For well-established healthcare organisations, it is recommended to integrate APPs into healthcare teams and support their practice through licensing, education and certification [16]. There is expected to be a shortage of primary care physicians in the United States (around 49,000) by 2030 because of the retiring workforce [17]. Increased patient load requiring medical attention coupled with workforce crisis threatens the future of primary care which is the backbone of the healthcare system and handles the majority of chronic disease management [17]. It is imperative to increase the supply of trained physicians and allied healthcare professionals to primary care. One plausible way to deal with this is to adopt a team-based approach where we induct PAs alongside nurse practitioners and physicians in the primary care setting. Around 21% of the 108,717 PAs have established themselves in primary care in contrast to 83.4% of the 222,000 licensed NPs. Keeping in mind the supply and demand mismatch there is estimated to be a 30% increase in the job opportunities for healthcare professionals in the coming decade [17]. With the induction of APPs into the workforce, issues surrounding communication skills can be predicted to arise within the teams. A solution that can address these issues can be simulation training. Another issue to address is the return of investment. Student/study loans were equivalent to $ 129,484 for both NPs and PAs. But the return of investment for NPs was slightly lower than the PAs, with a yearly wage of $ 89,960 for NPs and $ 90,930 for PAs on average [18]. Patients cohort seen by PAs or advanced practice registered nurses (APRNS) is more likely to include females, younger population and residents of rural communities [19]. They were less likely to exhibit markers of poor health. The patient cohort attended to by PA or APRN in an ancillary role included much sicker individuals [19]. It is also important to highlight that the non-medical practitioners (NMP) can be known by various names depending on the speciality they choose to focus on, some of the labels are PAs, surgical care practitioner, arthroplasty practitioner and laparoscopic nurse [20-21]. These many names to describe different roles can sometimes prove to be overwhelming for the patients and other healthcare practitioners. Building on from the success of the PA role in the US, in 2012, Health and Social care Act was introduced which brought about a change in the provision of health and social care services in the UK [22]. With a rising elderly population putting pressure on the health services it was decided to develop and focus on ‘mid level’ professionals like PAs and NPs [22]. PAs might be new to UK’s NHS but exist in a far more developed form in the US [22-25]. It is estimated that 200 PAs are rendering their services in the hospital across the UK employed at band 7 level to start with scope to promotion to Band 8a. Health Education England (HEE) aims to induct around 1000 PAs in primary care by 2020 [22,26]. ANP and PA roles are similar and dissimilar in a variety of ways. PAs follow a disease-centred medical model and ANPs follow a much more patient-centred model. ANPs additionally can prescribe whilst PAs cannot prescribe due to lack of regulation by a professional regulatory body but are maintained on a managed voluntary register [22,24,27-28].

PA’s role is very much comparable to ANP’s role and depending upon which healthcare they practice in PAs can diagnose, manage and prescribe under the supervision of senior clinicians/physicians. Because of their application in a variety of roles they are gaining popularity amongst healthcare policymakers.

In two of the referenced articles, we can find a similarity in the reasons why there was a need for the development of mid-level professionals like PAs and ANPs [17,22]. Policymakers had realised that they needed to act fast and effectively to address the expanding healthcare needs of the ever-growing elderly population especially in the primary care domain and their main response was to come up with the development and expansion of PA and ANP roles.

Doctors and PAs

The lack of primary care providers poses a major problem to the US healthcare system [29-32]. Healthcare is associated with a strenuous workload and due to clinical staffing level constraints and budgetary pressures healthcare systems have engineered new strategies to make sure the delivery of care [33-34]. One way to deal with these issues is the development of a mid-level practitioner who can streamline the provision of healthcare services by augmenting the role of physicians [33,35-36]. PAs can fill in this role better also known as PAs in the UK [33,37]. The PA profession came into being in the USA in the 60s and PAs are working across the spectrum of medical specialties [33,38]. Many doctors and members of the public recognise PAs and NPs as a core element in improving the performance of primary care [32]. Hence PA-primary physician partnership has been flourishing for over 5 decades now and continues to do so [32]. In accordance with local regulations, PAs are taught according to the medical model to manage patients independently, as consented by their supervisory doctor [33,38]. Healthcare systems in Australia, India, Canada, Germany and the Netherlands have tried to embrace the PA profession sticking to the same basic principles of the profession as introduced in the US [33]. US-trained PAs were introduced into the UK healthcare system in the first decade of this century (early to mid-2000s), with the first batch of locally trained PAs becoming the part of the workforce in the later part of the first decade (2009) [33,37,39-40]. The major difference between the UK trained and US-trained PAs is that UK trained PAs cannot prescribe as they are not yet regulated by a regulatory body [33,41]. With a greater emphasis on disease prevention and management of chronic diseases in the ever-growing elderly population in UK coupled with reduced trained staff availability in the primary care, it has been decided to induct PA workforce in the primary care to iron out these problems [33,42], with a government policy indicating to induct 100s of PAs within the primary care by the end of this decade [33,43]. It has been observed that ward rounds are less likely to be disrupted in the presence of PAs and they tend to increase doctor’s productivity by managing specific tasks in a more suitable way [30]. In the UK, it is still early days for this profession as it will take time to accustom the general public to this new role. Once more and more patients will get in contact with PAs and get educated about their roles and responsibilities it is anticipated that a trusting relationship will build up amongst the patients and the PAs as one exists between the patients and the GPs [33]. According to one of the studies, 27% of the respondent PAs were able to use point of care ultrasound with 62% of them optimistic about their skills [31]. The introduction of PA workforce into maxillofacial outpatient settings has allowed the surgeons to focus more on patient care whilst increasing the number of patients seen in the outpatient settings with better outcomes and this has translated into improved productivity and profits [44]. The primary reason for the difference in opinion between physicians and PAs has been related to differences in patient care and communication issues [29].

PAs in different healthcare systems are considered to be an extension of physicians who work under the supervision of an experienced clinician and execute management plans. Their role has shown to increase the efficiency of healthcare teams and improve patient safety. In certain healthcare systems they prescribe, but in others like in the UK, they are not allowed to prescribe as yet due to lack of regulation by regulatory bodies (for which legislation is underway).

Referenced articles (in this section of the discussion) advocate the application of PA role in different healthcare teams namely primary care, secondary inpatient care and secondary outpatient care [30,32,44]. Their utilisation in all these roles has proven to be worthwhile for the patients and teams alike.

Assistant Physicians and PAs

Assistant physicians (AP) in contrast to PAs are a new type of healthcare providers introduced in the US, who are primarily tasked to serve the patients in primary care settings in underprivileged rural areas. Qualifications wise, they have completed medical school and are ACGME/ECFMG-certified clinicians [45]. There is estimated to be a shortage of more than 45,000 doctors by 2025 in the US alone and thousands of doctors who apply via the matching system for residencies do not get the match [45]. So this creates a major shortage of the workforce that is to serve the ever-growing and ageing population; hence, in this climate, APs seem to suit the purpose of healthcare provision to the public in medically underserved areas [45]. Some forums advocate that the assistant/ associate physicians undergo far more rigorous medical training (including two years of clinicals) than compared to any of the other advanced practitioners, i.e, PAs, etc [46]. The state of Missouri in the US has been the first to legislate in favour of APs in allowing them to practice and serve the public in Missouri [47]. This legislation was signed by the Governor of Missouri in 2014. Requirements for licensure include; graduation from a recognised medical school, citizen or legally resident of the US, Passed USMLE step 1 and step 2, not accomplished completion of residency and proficiency in the English language [48]. Requirements for practicing include; Collaborative agreement with a sponsoring physician, 30-day internship with the sponsoring physician, work within 50-100 miles of the sponsoring physician and this physician must undertake a review of 10% of the Assistant physician’s charts [48]. Still in its early days but this profession has attracted its share of concerns and criticisms from circles of physicians about; length of the period of supervision and concerned authorities not taking the physician community into confidence about this new role. It has been reported that other states like Kansas, Arkansas and Utah have passed similar bills in support of APs [48]. According to Missouri Board of Registration of the Healing Arts in 2017, 99 assistant physicians were given license to practice [49]. Seven APs were from the US medical schools and 92 were international medical graduates. Twenty-five out of 92 assistant physicians had obtained a collaborative agreement and were serving in the areas where there was a shortage of healthcare staff [49]. Permissions have been given to APs to prescribe medications. The role of APs has attracted its fair share of criticism from different medical and regulatory circles and work is still undergoing to help this profession evolve in a safe environment for the patients.

The obvious similarity between the roles of APs and PAs is that these roles have been established to fill in the gaps that have developed in the healthcare workforce secondary to the shortage of trained doctors, both have to work under supervision of experienced doctors, and both can prescribe (note: PAs cannot still prescribe in certain healthcare systems). The differences that can be seen are the pathways each modality takes to licensure and practice, PAs do two- to three-year degree and have to pass a national exam (it can be different in different healthcare setups) to allow them to practice, whereas APs mostly are medical school graduates who have done their USMLE Step 1 and Step 2 but have not undergone a residency programme. Both PAs and APs are considered as an asset and future seems to be promising for both these professions.

Information available about the profession of AP is limited, and there is a scarcity of published scientific material on research search engines. The information derived from the assistantphysicianassociation.com speaks a lot in favour of this profession and depicts it as a valid solution for the staff shortages in the healthcare teams and there are articles as published on the www.medscape.com where certain concerns have been raised about this new profession indicating a need for further regulatory controls and professional training for APs. Table 1 summarises a few important points.

**Table 1:** Important points to consider US, United States; UK, United Kingdom; APP, advanced practice providers; NP, nurse practitioner; PA, physician assistant/physician associate; ROI, return of investment [16-18], [30], [32-33], [44]

Author	Year	Country	Study's Focus	Summary
Waldrop et al.	2019	US	Support and Unification of Advanced Practice Providers	Healthcare institutions should support practice of APPs using Professional development activities that helps them maintain their License to practice.
Faza et al.	2018	US	Effectiveness of NPs and PAs	In Face of a major shortage of healthcare professionals, the introduction of NPs and PAs into the healthcare system will prove to be a very successful strategy
Craig et al.	2017	US	Return on investment of NPs and PAs	PAs have a slightly higher ROI compared with NPs
Hascall et al.	2018	US	PAs and ward rounds	PAs improve productivity and efficiency in addition to reducing interruptions in the ward rounds
Coplan, Smith, & Cawley	2017	US	PAs in primary care	PA and physician partnership model is set to flourish in the current environment as healthcare faces shortage of primary care physicians
Resnick et al.	2016	US	PAs in outpatient clinic settings	Introduction of PAs in the outpatient setting will improve the flow of patients allowing more patients to be seen in outpatient clinic settings
Halter et al.	2017	UK	Patient experience of PA in primary care settings	Patient experience after consultations with PAs has largely being positive but the patient will like to know more about the role of PAs.

PAs as preceptors and medical educators

With a skill mix that PAs possess they should be considered as an asset towards medical education. PAs further specialising in certain field of medicine and surgery can add extra value to any taught medical programme. PAs can not only teach general medical topics but can work closely with physicians in conducting simulations. PAs have been seen as a valued member of the examiner teams by different organisations where they set high level of standards in ensuring that only safe and proficient candidates pass the exams and join the prestigious medical fraternity.

Recent update: General Medical Council (UK) set to regulate PA profession in the United Kingdom

In one of the most recent updates, it has been announced that the General Medical Council (UK) is going to regulate the PA profession in the UK [50]. This can mean that there is a strong possibility that PAs in the UK might in the future (once regulated by the GMC) be able to prescribe medications and request investigations involving ionizing radiations, but this can not be ascertained at this point of time. GMC is working closely with the Department of Health and Social Care (UK) to determine costs and timescales. In our opinion, this can be a ground-breaking development for the PA profession in the UK, which might turn out to be a light bearer for other healthcare systems in the Europe, Australia and the Middle East. This step will also lead to ensuring higher standards of care provision, training and patient safety [50].

Limitations

Several limitations became clear whilst reviewing the published information about PAs, their role definition and curriculum. Most of the data with regard to this profession originate from the USA as this profession has most evolved in the USA. There is some contribution to the literature from Europe more of it originating from the UK where this profession is still trying to find its footings. But there is a scarcity of literature coming from the rest of the world where there is much less known about this profession. Our literature review aims to provide insight into this profession, and we hope our word reaches out to such places where policymakers start to realise the importance of the role of PAs in healthcare and its economics. But we also realise that the on-ground realities in other healthcare systems might be different and their outlook and application of the insight provided in our review article might not be similar to the systems where this role has or is flourishing. Also, we will like to indicate that we found it difficult to locate scientifically peer-reviewed articles with regard to the role of an AP.

## Conclusions

Our review article defines various roles often taken up by the PAs and how they can integrate within different healthcare teams in a multidisciplinary environment. We also aimed to compare the roles undertaken by the PAs with other members of the healthcare team. We found out that PAs have shown to work under a diverse array of circumstances and conditions ranging from general primary care sector to specialised tertiary care centres. It demonstrates that PAs can acquire a wide array of skills which helps them find their footings in diverse healthcare environments. We also found out that different healthcare systems around the world regulate this profession differently which has a bearing on how these professionals perform within those healthcare systems. We think that the PA profession has evolved over the period of decades and now in its current state ready to be introduced to many other healthcare systems of the world (in addition to western healthcare systems). PAs are a force multiplier within the healthcare sector and can be seen as a valid solution to staff shortages faced by the healthcare systems around the world. Understanding the roles is the first and most important step of the process as it helps with the application and execution phase of the process. It will help other members of the team understand how to integrate and work along with PAs to maximise team’s potential. It will further help the wider community to understand about the PA profession and help them clarify a lot of their misunderstandings. We recommend further studies looking into different aspects of the role of a PA that could further provide our readers with clarity with regard to PAs.

## References

[1] (2019). Physician Assistant Stats, Data, and Demographics. https://www.thepalife.com/physician-assistant-stats.

[2] Kevin C. Lohenry, Anthony Brenneman, Constance Goldgar (2017). Entrustable professional activities: a new direction for PA education?. J Physician Assist Educ.

[3] Englander R, Cameron T, Ballard A (2013). Toward a common taxonomy of competency domains for the health professions and competencies for physicians. Acad Med.

[4] Craig CK, Holmes JH, Carter JE (2017). Return on investment of advanced practice medical degrees: NPs vs. PAs. J Physician Assist Educ.

[5] (2019). State-level projections of supply and demand for primary care practitioners: 2013-2025. https://bhw.hrsa.gov/sites/default/files/bhw/health-workforce-analysis/research/projections/primary-care-state-projections2013-2025.pdf.

[6] 6). The Complexities of (2019). The complexities of physician supply and demand 2017 update: projections from 2015 to 2030. https://aamc-black.global.ssl.fastly.net/production/media/filer_public/31/13/3113ee5c-a038-4c16-89af-294a69826650/2019_update_-_the_complexities_of_physician_supply_and_demand_-_projections_from_2017-2032.pdf.

[7] American College of Physicians (2019). The impending collapse of primary care medicine and its implications for the state of the nation's health care. https://www.acponline.org/acp_policy/statements/impending_collapse_of_primary_care_medicine_and_its_implications_for_the_state_of_the_nations_health_care_2006.pdf.

[8] Division of Advocacy and Health Policy (2006). A growing crisis in patient access to emergency surgical care. Bull Am Coll Surg.

[9] Institute of Medicine (2007). Emergency Medical Services: At the Crossroads.

[10] Sklar DP (2013). How many doctors will we need? A special issue on the physician workforce. Acad Med.

[11] (2017). National Commission on Certification of Physician Assistants. 2015 Statistical Profile of Certified Physician Assistants by Specialty: An Annual Report of the National Commission on Certification of Physician Assistants. http://www.nccpa.net/research.

[12] Hooker RS, Brock DM, Cook ML (2016). Characteristics of nurse practitioners and physician assistants in the United States. J Am Assoc Nurse Pract.

[13] de Lusignan S, McGovern AP, Tahir MA (2016). Physician associate and general practitioner consultations: a comparative observational video study. PLoS One.

[14] Constance Goldgar, Ed Michaud, Nguyen Park (2016). Physician assistant genomic competencies. J Physician Assist Educ.

[15] Rolls J, Keahey D (2016). Durability of expanded physician assistant training positions following the end of health resources and services administration expansion of physician assistant training funding. J Physician Assist Educ.

[16] Waldrop J, Heinl V, Mestas L (2019). Systematically building a model to support and unify advanced practice providers. J Nurs Adm.

[17] Faza NN, Akeroyd JM, Ramsey DJ (2018). Effectiveness of NPs and PAs in managing diabetes and cardiovascular disease. JAAPA.

[18] Craig CK, Holmes JH, Carter JE (2017). Return on investment of advanced practice medical degrees. JAAPA.

[19] Everett CM, Morgan P, Jackson GL (2016). Patient characteristics associated with primary care PA and APRN roles. JAAPA.

[20] Abraham J, Whiteman B, Coad J, Kneafsey R (2016). Development and implementation of non-medical practitioners in acute care. Br J Nurs.

[21] Moorthy R, Grainger J, Scott A, Powles JW, Lattis SG (2006). Surgical care practitioner - a confusing and misleading title. The Bulletin of the Royal College of Surgeons of England.

[22] Peate I (2016). The physician’s associate. Br J Nurs.

[23] Buchanan J, O'May F, Ball J (2007). New role, new country: introducing US physician assistants to Scotland. Hum Resour Health.

[24] Drennan VM, Halter M, Joly L (2015). Physician associates and GPs in primary care: a comparison. Br J Gen Pract.

[25] Global Health Workforce Alliance (2019). Mid-level health workers for delivery of essential health services. Global Health Workforce Alliance.

[26] Matthews-King A (2019). Physician associate training places to expand by 220% next year. http://www.pulsetoday.co.uk/your-practice/practice-topics/employment/physician-associate-training-places-to-expand-by-220-next-year/20031163.article.

[27] (2019). Physician Associate Managed Voluntary Register. https://www.plymouth.ac.uk/uploads/production/document/path/8/8121/CCF-27-03-12-for-PAMVR.pdf.

[28] Ritsemeh TS 2015 United Kingdom Association of Physician Assistants Census Results. http://tinyurl.com/nf9pvgz.

[29] Bochatay N, Bajwa NM, Cullati S (2017). A multilevel analysis of professional conflicts in health care teams: insight for future training. Academic Medicine.

[30] Hascall RL, Perkins RS, Kmiecik L (2018). PAs reduce rounding interruptions in the pediatric intensive care unit. JAAPA.

[31] Rizzolo D, Krackov R (2018). PA use of point-of-care ultrasound. JAAPA.

[32] Coplan B, Smith N, Cawley JF (2017). PAs in primary care: current status and workforce implications. JAAPA.

[33] Halter M, Drennan VM, Joly LM (2017). Patients’ experiences of consultations with physician associates in primary care in England: A qualitative study. Health Expectations: An International Journal of Public Participation in Health Care and Health Policy.

[34] World Health Organisation (2019). Health Workforce 2020. http://www.who.int/hrh/documents/strategy_brochure2014/en/.

[35] World Health Organisation and Global Health Workforce Alliance (2019). Mid‐level health providers a promising resource to achieve the health millennium development goals. World Health Organisation and Global Health Workforce Alliance.

[36] Sibbald B, Shen J, McBride A (2004). Changing the skill‐mix of the health care workforce. J Health Serv Res Policy.

[37] (2019). Faculty of Physician Associates at the Royal College of Physicians: Frequently Asked Questions. https://www.fparcp.co.uk/about-fpa/faqs.

[38] (2019). Bureau of Labour Statistics. Occupational Outlook Handbook. http://www.bls.gov/ooh/healthcare/physician-assistants.htm.

[39] Woodin J, McLeod H, McManus R (2019). Evaluation of US‐trained Physician Assistants working in the NHS in England. https://www.birmingham.ac.uk/Documents/college-social-sciences/social-policy/HSMC/publications/2005/Evaluation-of-US-trained-Physician-Assistants.pdf.

[40] Farmer J, Currie M, Hyman J (2011). Evaluation of physician assistants in National Health Service Scotland. Scott Med J.

[41] Ross N, Parle J, Begg P, Kuhns D (2012). The case for the physician assistant. Clin Med.

[42] (2019). The future of primary care: creating teams for tomorrow. https://www.hee.nhs.uk/sites/default/files/documents/The%20Future%20of%20Primary%20Care%20report.pdf.

[43] Hunt J (2019). New deal for general practice. https://www.gov.uk/government/speeches/new-deal-for-general-practice.

[44] Resnick C, Daniels K, Flathsporn S, Doyle M, Heald R, Padwa BL (2016). Physician assistants improve efficiency and decrease costs in outpatient oral and maxillofacial surgery. J Oral Maxillofac Surg.

[45] (2019). Association of Medical Doctor Assistant Physicians. https://assistantphysicianassociation.com/.

[46] (2019). Qualifications of an assistant physician. https://assistantphysicianassociation.com/projects.

[47] Assistant Physicians and Associate Physicians meet the (2019). Experience meets deficit. https://assistantphysicianassociation.com/partners.

[48] (2019). New assistant physician licensure program raises concern. https://www.medscape.com/viewarticle/903840.

[49] (2019). Study reveals troubling test scores of assistant physicians. https://www.aafp.org/news/practice-professional-issues/20181109asstphys.html.

[50] (2019). GMC to regulate two new associates roles. https://www.gmc-uk.org/news/news-archive/gmc-to-regulate-two-new-associates-roles---pas-and-aas.

